# Research on the oil retention effect of pneumatic oil barriers under current and wave action

**DOI:** 10.1371/journal.pone.0322390

**Published:** 2025-05-08

**Authors:** Hao Liu, Hongyuan Sun, Bo Jiao, Haihua Lin, Guoxing Wang

**Affiliations:** College of Naval Architecture and Port Engineering, Shandong Jiaotong University, Weihai, China; NED University of Engineering and Technology, PAKISTAN

## Abstract

The pneumatic oil barrier is a novel device that intercepts oil spills by releasing high-pressure gas underwater to form a curtain of bubbles. However, its performance can be affected by both internal factors and external influences like water flow and waves, leading to varying degrees of oil interception failure and reduced effectiveness. This study combines physical experiments with numerical simulations to analyze the impact of nozzle parameters, arrangement, flow velocity, and wave effects on oil interception loss and effective containment distance under uniform flow and wave conditions. The findings establish relationships between these factors and interception efficiency, revealing reasons for changes in effective containment distance through flow field analysis. The research indicates that the pneumatic oil barrier is effective for floating oil with flow velocities below 0.15 m/s; the optimal nozzle diameter is 1.5 mm, as increasing it affects the stability of horizontal flow and oil layers. Additionally, a dual-pipe arrangement produces a wider and more stable horizontal flow on the water surface, enhancing oil interception effectiveness, while waves can destabilize oil layers, reducing effective containment distance.

## Introduction

As a new type of oil containment device, the pneumatic oil barrier operates by releasing high-pressure gas underwater to create a curtain of bubbles. When the bubbles rise to the surface, they cause the surrounding water to move upward, creating a horizontal flow at the surface. Additionally, when the bubbles burst at the surface, they produce upwellings. The pneumatic oil barrier uses these upwellings and the horizontal flow, which is in the opposite direction of the oil spill dispersion, to control the spread of the oil. [1,2] Compared to traditional oil barriers, the pneumatic oil barrier is deployed underwater, does not interfere with navigation, and is easier to start, clean, recover, and maintain. [3] It is also effective in containing burning oil, demonstrating significant potential in engineering applications. [4,5]

Since the U.S. Coast Guard first proposed the oil containment capability of bubble curtains in the last century, scholars have conducted extensive research on the reliability of pneumatic oil barriers. [6] Early research [7] suggested that oil containment failure is related to the flow velocity of horizontal currents. Some researchers believed that, under flowing water conditions, the maximum flow velocity of the surface horizontal current is related to factors such as oil layer thickness and oil-water density ratio. These factors exhibit a ratio at the onset or occurrence of failure, which is a constant termed the critical failure value. However, Delvigne [8] found that oil containment failure, known as the escape phenomenon, can also occur at low flow velocities. He noted that the occurrence of such escape phenomena increases with the flow velocity of horizontal currents. He argued that the flow field around pneumatic oil barriers during oil containment is highly complex, making it impossible to define a critical state for oil containment failure. Therefore, analyzing the escape velocity of oil could be crucial to studying the effectiveness of pneumatic oil barriers. However, both physical and field experiments face limitations in accurately measuring oil containment losses, hindering further research into oil containment effectiveness. [6,9] Consequently, current research lacks a focus on analyzing oil containment losses to assess the effectiveness of pneumatic oil barriers. With the introduction of bubble curtain simulation software by Pan et al. [10–14], this challenge may have a potential solution. Nonetheless, Xie Senshen et al. [4,13,15] combined physical experiments and numerical simulations to study the oil containment performance under shear flow conditions, but they only analyzed changes in flow field distribution and the location where the oil spill was blocked. They found that when the blocked oil is further away from the horizontal position of the pneumatic oil barrier’s exhaust pipe, the effectiveness and reliability are improved. Although their work did not address the analysis of oil containment losses, it introduced a new method for evaluating oil containment reliability. [16] Similarly, Wu Lin conducted numerical simulation analyses of pneumatic oil barriers but only observed the leakage phenomena during containment failure. [17] From the above studies, it is evident that current research lacks monitoring and quantitative studies on oil spill losses. Another shortcoming is that most of the above studies were conducted in static or flowing water environments, while research on the performance and effectiveness of pneumatic oil barriers under wave conditions is still scarce. [1] Some studies on the application of bubble curtains as breakwaters suggest that waves may cause bubble curtains to deviate in shape, compromising their integrity and potentially leading to oil containment failure. [18–22] Furthermore, the combined effects of waves and bubble curtains increase water surface disturbances, which may impact the stability of the oil layer. These factors may explain the limited research on wave-influenced environments. However, since waves are common in open water areas such as oceans and lakes, incorporating wave effects is essential for studying the oil containment performance of pneumatic oil barriers.

In summary, most scholars have primarily focused on the correlations between influencing factors at the critical failure point of pneumatic oil barriers. Although a few recent studies have used the concept of effective oil containment distance to conduct one-sided quantitative analyses of oil containment reliability, they often overlook the crucial factor of oil loss. (The effective oil containment distance refers to the horizontal distance between the pneumatic oil barrier’s exhaust pipe and the location where the oil spill is blocked, i.e., the leading edge of the oil layer, as shown in Fig 2(a). It is worth noting that when the blocked oil appears downstream of the exhaust pipe (no leakage occurs), the horizontal distance from the exhaust pipe to the blocked oil position is considered negative, as illustrated in Fig 2(b).). Moreover, there remains a lack of research on the impact of waves on the oil containment performance of pneumatic oil barriers.

**Fig 1:**
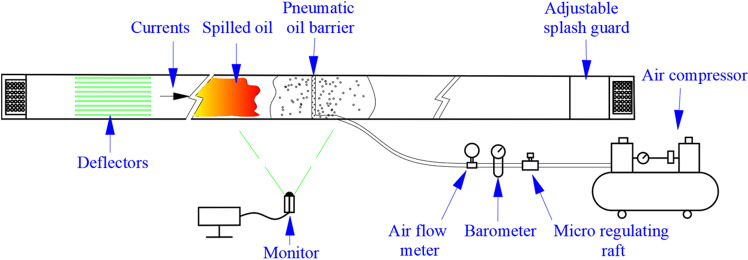
Experimental setup diagram (Top view).

Therefore, this study will systematically investigate the oil containment performance of pneumatic oil barriers under uniform flow and wave conditions by analyzing oil loss and considering changes in effective oil containment distance. Effective oil containment distance (S) is used as an auxiliary metric to analyze the oil containment performance of pneumatic oil barriers. A greater effective oil containment distance provides a larger buffer against disturbances such as bubble curtain deviation, water surface agitation, and other external factors, thereby improving oil stability. Consequently, a larger effective oil containment distance indicates better oil containment performance and greater resistance to interference. Oil loss refers to the amount of oil that escapes beneath or over the pneumatic oil barrier (assuming that the floating oil does not evaporate or dissolve in water, given current technological limitations in intercepting volatile and water-soluble substances during real oil spill incidents).

To simplify the expression of oil loss, this study uses the dimensionless oil spill volume loss rate E(s^-1^), which is defined as the volume of oil passing through the pneumatic oil barrier per unit width per second (m^3^·m^-1^·s^-1^) divided by the total volume of oil per unit width (m^3^·m^-1^). The product of actual oil spill volume (m^3^) and the dimensionless oil spill volume loss rate under identical conditions can predict the rate of oil volume passage in actual spill scenarios. According to the above analysis, the velocity of surface horizontal flow is influenced by nozzle diameter, nozzle angle, and air supply rate, with nozzle diameter and air supply rate having greater impacts than nozzle angle. [23] Additionally, the nozzle arrangement may affect the thickness of horizontal flow, while waves may influence the stability of the bubble curtain. Considering these factors, this study will investigate the oil containment performance of pneumatic oil barriers by examining nozzle parameters (diameter and angle),[17] arrangement methods, flow velocity, and wave parameters (wave height and period). [24] Furthermore, the flow field and vorticity under different parameter variations will also be analyzed to provide reference data for the practical application of pneumatic oil barriers.

### The physical experiment platform

**Fig 2:**
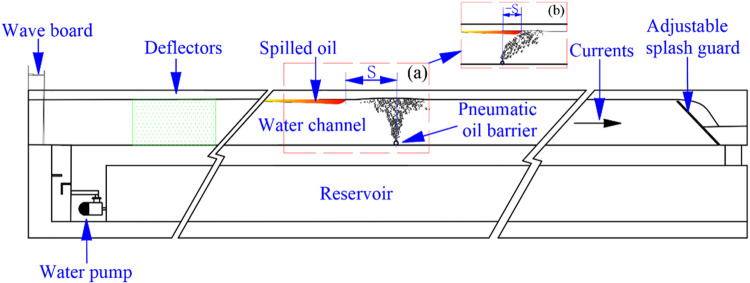
Experimental setup diagram (Front view).

The physical experiments were conducted in a recirculating water tank. The layout of the tank and experimental environment is shown in Fig 1 (top view) and Fig 2 (side view). The tank is situated above a reservoir, which provides the water source, and an electric pump supplies the flow power. The variable-speed pump can adjust the water flow velocity, and the experimental water exits the tank at the end and returns to the reservoir. The tank, reservoir, and electric pump form a dynamic, circulating flow system. Considering that the walls of the tank may produce boundary effects that could influence the experimental results, we treated the tank walls prior to the experiment. The walls were coated with a hydrophobic material commonly used for anti-fog and anti-rain applications in automobiles. This coating adheres to the surface of the tank walls, providing excellent hydrophobic properties that significantly reduce friction between the water flow and the walls. This minimizes the impact on oil diffusion and the shape of the bubble curtain.

Additionally, the water flow velocity in the experiment was scaled down. By reducing the flow velocity, the friction between the fluid and the tank walls is further minimized, helping to alleviate local turbulence and reduce the nonuniformity and instability of the boundary layer. At the inlet of the tank, a diversion plate was installed, and the deployment location of the pneumatic oil barrier was set sufficiently far from the tank’s inlet and outlet. This arrangement helps to reduce turbulence caused by boundary effects and ensures uniform flow in the experimental observation area.

The tank is divided into two functional zones: a wave-generating system at the front end and a wave-absorbing system at the rear end. The single-panel paddle wave generator, located at the front end, operates by inputting frequency and oscillation amplitude parameters via a wave generation program interface. It uses a crank-rocker transmission system to control the paddle’s oscillation at a constant speed or periodic motion. The paddle’s amplitude and period determine the wave height, wavelength, and period. The single-panel paddle wave generator produces regular waves and can simulate certain ocean conditions to a limited extent. However, it has significant limitations in replicating the complexity of real marine environments. Natural ocean waves are influenced by multiple factors, such as seabed topography, wind speed, and tidal forces, resulting in highly complex nonlinear and stochastic wave characteristics that are difficult for paddle wave generators to mimic. Research on the oil containment performance of pneumatic oil barriers under wave conditions is still in its early stages. Regular waves were selected for this study due to their higher controllability and comparability. At the rear end of the tank, the wave-absorbing system comprises a sloped wave absorber designed at a specific angle to interact effectively with the waves. This setup absorbs wave energy and prevents residual waves caused by wave reflections from interfering with the experimental results. A capacitive intelligent wave gauge is positioned in front of the pneumatic oil barrier to monitor wave parameters near the barrier. These parameters are transmitted to a PC for analysis, as illustrated in Fig 4(a) and (b).

**Fig 3:**
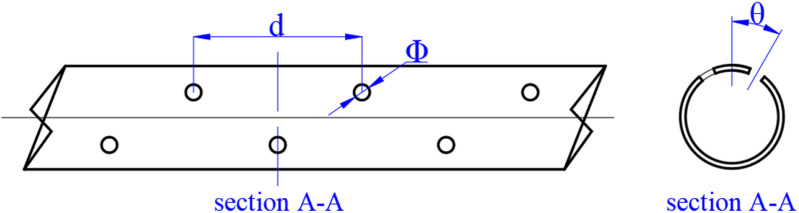
Schematic diagram of exhaust pipe dimensions.

The nozzles of the pneumatic oil barrier model are uniformly distributed on both sides of the exhaust pipe at a specific angle. During the experiment, the effects of nozzle diameter and angle on oil containment effectiveness are considered. Therefore, models with different nozzle diameters were made according to the experimental conditions, as shown in Fig 3, where Φ represents the nozzle diameter, θ represents the nozzle angle, and d represents the distance between nozzles in the same row. The appropriate nozzle diameter and angle have significant effects on bubble size, distribution density, and the stability of the oil layer. The nozzle diameter determines the gas outlet velocity and bubble size. A larger nozzle diameter results in a lower gas outlet velocity, producing larger but fewer bubbles. Smaller bubbles contribute to a more stable bubble curtain but may disperse more in flowing water, reducing oil containment effectiveness at lower horizontal flow velocities. On the other hand, excessively large bubbles create greater disturbances to the water surface when they burst, affecting the stability of the oil layer and reducing containment efficiency. Compared to previous studies that examined three nozzle diameters, this study selects five nozzle diameters: 1 mm, 1.5 mm, 2 mm, 2.5 mm, and 3 mm. These selections allow for a more precise determination of the optimal diameter for effective oil containment. The nozzle angle influences the direction of airflow and the distribution of bubbles. An appropriate nozzle angle helps expand the influence area of the bubble curtain, forming an effective barrier. This study considers five nozzle angles: 0°, 30°, 45°, 60°, and 90°, aiming to identify the most suitable angle for optimal performance.

**Fig 4:**
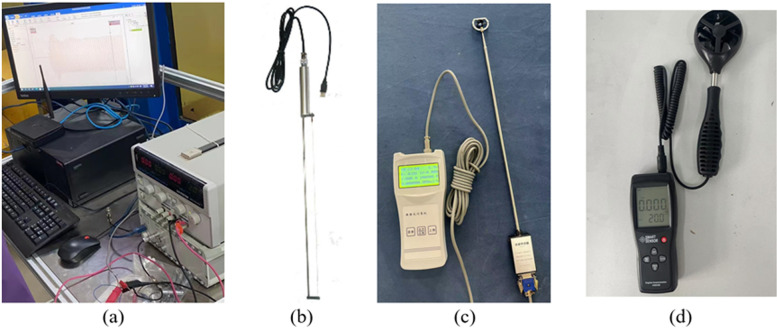
Experimental measurement equipment.

The pneumatic oil barrier is supplied with high-pressure gas from an air compressor. The air compressor can provide a supply pressure of 0.8 MPa and a flow rate of 380 L/min·m, meeting the experimental requirements. An air flow meter and pressure gauge are installed on the gas supply pipe connecting the pneumatic oil barrier to the air compressor, allowing measurement of instantaneous and average gas flow rates and supply pressure. An air flow control valve is used to adjust the experimental gas flow rate and pressure. To ensure the accuracy of water velocity in the experimental area, water velocity meters were placed near the pneumatic oil barrier. The water velocity meters have a measurement error of 1.5%, as shown in Fig 4(c). In the observation area, both a fixed and a movable camera system are deployed to record the oil containment phenomena of the pneumatic oil barrier in real-time during the experiment. The results of the physical experiments are obtained as averages from five measurements, ensuring accuracy in the results.

The physical experiments were conducted in a controlled indoor laboratory environment. The temperature and humidity of the laboratory were precisely regulated using thermostatic equipment and humidity controllers to ensure environmental conditions remained within the specified range. During the experiments, the indoor temperature was maintained at 20±2°C, and the humidity was controlled at 40%±4%RH. The water temperature used in the experiments was continuously monitored and recorded to ensure stability. Additionally, the fully enclosed experimental setup prevented interference from external wind. Fluctuations in indoor temperature, water temperature, or wind conditions could affect experimental results. For instance, excessively high or low temperatures might alter the viscosity of the experimental oil, while variable wind speeds could interfere with the diffusion direction of the floating oil.

Since the effects of temperature and wind speed on the oil containment performance of the pneumatic oil barrier are not the focus of this study, only their conditions were controlled without further investigation into their impacts. The equipment used for environmental control and measurement is shown in Fig 4(d). The image shows the instruments used to measure wind speed and environmental temperature, with a wind speed measurement error of 3% and a temperature measurement accuracy of ±2°C. Based on the accuracy and errors listed in **Table 1** and the measurements taken during the experiments, uncertainty and error analysis were conducted for the physical experiments. Using the single-sample error calculation method, [25–27] the relative error of the experimental results was determined. The errors for horizontal flow velocity and effective oil containment distance were calculated to be ±0.104% and ±2.145%, respectively.

**Table 1:** Technical specifications of measuring instruments.

Measuring Equipment		Accuracy	Range	Error (%)
Capacitive Intelligent Wave Gauge		±0.1mm	0~0.5m	0.2
Gas Flow Meter		±(2.0+0.5FS)%	0~250L/min	0.5
Water Velocity Meter		±0.003m/s	0.01~4.00m/s	1.5
Pressure Gauge		±0.004MPa	–0.1~60Mpa	0.25
Split-Type Wind and Temperature Measuring Instrument	Temperature Measurement	±2°C	0~45°C	0.2
	Wind Speed Measurement	±3%rdg±0.1	0~45m/s	0.3

In this study, the Reynolds similarity theorem was used to select the experimental oil and water flow speed (v_l_), with a scaling factor of λ. The experimental oil chosen is soybean oil with a viscosity of 92.9 cSt, corresponding to an industrial oil with a viscosity of 482.688 cSt when scaled by λ^3/2^. **Table 2** lists the density, viscosity, and surface tension of common industrial oils used in offshore transportation and at oil ports, such as Kuwait crude oil, light lubricating oil, and heavy oil. Although industrial oils such as soybean oil and crude oil differ in chemical composition, the scaled properties of soybean oil are similar to those of light oils (such as light lubricating oil) in terms of viscosity, density, and surface tension. Therefore, soybean oil can be used to simulate the leakage behavior of light oils (e.g., diesel and gasoline). Moreover, unlike light industrial oils, soybean oil does not have volatility, making it more suitable for observation and study. Compared to other high-viscosity industrial oils, soybean oil has lower surface tension, which may result in stronger spreading on the water surface. However, in physical experiments, it is easier to observe phenomena like oil diffusion and entrainment failure, and it is more controllable, providing meaningful comparisons for the study of pneumatic oil barriers.

**Table 2:** Comparison of physical properties between soybean oil and common industrial oils in offshore transportation and oil ports.

Name	Density (15°C)	Viscosity (15°C)	Surface Tension
Soybean Oil	0.91–0.93 g/cm^3^	92.9 cSt	30–35 mN/m
Kuwait Crude Oil	0.86–0.88 g/cm^3^	10–14 cSt	35–50 mN/m
Light Lubricating Oil	0.85–0.88 g/cm^3^	100–300 cSt	30–35 mN/m
Heavy Oil	0.85–0.88 g/cm^3^	1,000–10,000 cSt	40–60 mN/m

For observation purposes, soybean oil was dyed with the oil-soluble dye Sudan II (chemical formula C_18_H_16_N_2_O), which dyes the soybean oil red while not affecting the color of the water. Water flow speed significantly influences the bubble curtain shape, oil layer movement speed, and the stability of the water body. This experiment quantitatively studied the oil containment effectiveness under different water flow conditions. The scaling factor for water flow speed is λ^1/2^, with an experimental water flow speed of 0.1 m/s, corresponding to a real water flow speed of 0.17 m/s. The scaling calculation method is detailed in Delvigne’s work. [8]

## Numerical simulation

### Continuity equation

The simulation software utilized in this study is the commercial software ANSYS Fluent, which utilizes two-dimensional modeling for numerical simulation. [28] The model utilizes the Volume of Fluid (VOF) method combined with the Discrete Phase Model (DPM) for calculations. [12,29] In the numerical simulation of pneumatic oil barriers, it is necessary to simulate the interactions between the three phases: air, oil layer, and water. The VOF method is primarily used to track the interfaces between the air, water, and oil phases and describe their interactions. The DPM method is suitable for particle phases like bubbles and is used to simulate the movement and dynamics of bubbles in water, capturing their rupture on the water surface and the stability of the pneumatic oil barrier. The coupling of these two methods allows for a more comprehensive and accurate simulation of the interactions between the bubble curtain and the oil-water interface during the operation of the pneumatic oil barrier.

Although the combination of the VOF and DPM methods may have some potential limitations, especially in handling interactions between bubbles and the compressibility of bubble volume, the pneumatic oil barrier mainly relies on the bubble curtain to alter the local density and generate horizontal flow on the water surface to intercept the spread of oil. Since the experimental setup has a relatively shallow water depth, the impact of bubble compressibility on the research question is minimal. Later, appropriate mesh division and time step selection will be used to ensure the accuracy and stability of the numerical simulation. In the calculations, the air, water, and oil are treated as three phases, denoted as gas phase (*g*), liquid phase (*l*), and oil phase (*o*), respectively. The volume fractions of the three phases are represented as α_g_, α_l_, and α_o_, satisfying the constraint: *α*_*g*_ + *α_l_* + *α_o_* = 1.

Since the fluid domain is relatively small and the water depth of the scaled pneumatic oil barrier deployment is 0.3m, bubbles typically move under low-pressure conditions. The pressure-induced bubble volume changes are minimal, and the effect of bubble compressibility on the simulation results is small. Especially, the impact of bubble volume changes on horizontal flow, surface disturbance, and oil layer stability is negligible. To simplify the calculation and improve simulation efficiency, the three phases are treated as incompressible fluids in the simulation. The relationships between density (*ρ*) and viscosity (*μ*) among the three phases are expressed as follows:


$$\rho =\alpha_{g}\rho_{g}+\alpha_{l}\rho_{l}+\alpha_{o}\rho_{o}$$ (1)

$$\mu =\alpha_{g}\mu_{g}+\alpha_{l}\mu_{l}+\alpha_{o}\mu_{o}$$ (2)

The Volume of Fluid (VOF) model is utilized to simulate and calculate different immiscible fluids by solving a set of momentum equations and tracking the volume fractions of each incompressible fluid in the domain. The continuity equation can be expressed as:


$$\frac{\partial \alpha_{q}}{\partial \text{t}}+\frac{\partial (u\alpha_{q})}{\partial \text{x}}+\frac{\partial (w\alpha_{q})}{\partial y}=0,q=g,l,o$$ (3)

### Momentum equation

The momentum equation for viscous fluid is represented using the Navier-Stokes equation, as follows:


$$\begin{array}{l} \frac{\partial }{\partial t}(\rho \bar{u_{i}})+\frac{\partial }{\partial x_{j}}(\rho \bar{u_{i}}\bar{u_{j}})=-\nabla p+\nabla \left[\mu (\frac{\partial \bar{u_{i}}}{\partial x_{j}}+{\frac{\partial \bar{u_{j}}}{\partial x_{i}}}^{T})\right]\\ -\nabla (\rho \bar{u_{i}^{\prime }u_{j}^{\prime }})+\rho g_{i}+F_{i}^{c}+\sigma \frac{K_{g}\nabla \alpha_{g}}{\frac{1}{2}(\rho_{g}+\rho_{l})} \end{array}$$ (4)

The equation includes $F_{i}^{c}$, which represents the force generated during the coupling of multiple bubbles, and the last term represents the surface tension of the water phase, $K=\nabla (\frac{\nabla \alpha_{q}}{\left|\nabla \alpha_{q}\right|})$, where $K_{g}=-K_{l}$, $\nabla \alpha_{g}=-\nabla \alpha_{l}$.

### Turbulence model

To address the influence of convection and diffusion on turbulent flow, a suitable turbulent model needs to be selected. The turbulence model chosen for this study is the RNG k-ε model. This model is similar in form to the standard RNG model but includes an additional term in its ε equation to improve accuracy for high-speed flows. The equations for the RNG k-ε model are as follows:


$$\{\begin{array}{c} \begin{array}{l} \frac{\partial }{\partial \text{t}}(\rho k)+\frac{\partial }{\partial x_{i}}(\rho ku_{i})=\frac{\partial }{\partial x_{j}}(\alpha_{k}\mu_{e}f\frac{\partial k}{\partial x_{j}})+G_{k}\\ +G_{b}-\rho \varepsilon -Y_{M}+S_{k} \end{array}\\ \begin{array}{l} \frac{\partial }{\partial \text{t}}(\rho \varepsilon )+\frac{\partial }{\partial x_{i}}(\rho \varepsilon u_{i})=\frac{\partial }{\partial x_{j}}(\alpha_{k}\mu_{e\text{ff}}\frac{\partial \varepsilon }{\partial x_{j}})+C_{1\varepsilon }\frac{\varepsilon }{k}(G_{k}\\ +C_{3\varepsilon }G_{b})-C_{2\varepsilon }\rho \frac{\varepsilon^{2}}{\text{k}}-R_{\varepsilon }+S_{\varepsilon } \end{array} \end{array}$$ (5)

In the previous equation, *G_k_* represents the turbulence kinetic energy generated by the velocity gradient of laminar flow, while *G_b_* represents the turbulence kinetic energy generated by buoyancy. Compared to the standard RNG model, the RNG k-ε model can more accurately simulate the effects of vortices on turbulence. [14] For the high Reynolds number k−ε model, it is typically required that 30<y+ <300. In this study, the lower limit of y+ is 42.

### Fifth-order stokes model

In this study, a sinusoidal regular wave with a finite amplitude is used. However, the bubbles from the pneumatic oil barrier also introduce nonlinear effects on the regular wave when they burst at the water surface. Therefore, the fifth-order Stokes model is employed to describe the propagation of water waves. This model accounts for nonlinear effects and viscous resistance, providing a more accurate prediction of wave behavior. [30]

For the fifth-order Stokes model, the generation of waves can be achieved by determining the fluid velocity at the boundaries in the *x* and *z* directions and the instantaneous wave height at the water surface. The boundary velocity for wave generation and the instantaneous wave height at the water surface are given by:

In the *x* direction, the velocity is given by:


$$u_{x}=c\sum_{n=1}^{\text{j}}\text{n}\lambda_{\text{n}}\cosh\left[nk(z+h)\right]\times \cos\left[n(kx-\omega t)\right]$$ (6)

In the *z* direction, the velocity is given by:


$$u_{z}=c\sum_{n=1}^{\text{j}}\text{n}\lambda_{\text{n}}\sinh\left[nk(z+h)\right]\times \sin\left[n(kx-\omega t)\right]$$ (7)

The instantaneous wave height at the water surface is given by:


$$\eta =\frac{1}{k}\sum_{n=1}^{5}\lambda n\cos\left[n(kx-\omega t)\right]$$ (8)

where: *ω* is the angular frequency; *x* is the horizontal position; *t* is the time; *k* is the wave number. In the expression, the coefficients are as follows:


$$\begin{array}{l} \lambda_{1}=\lambda \\ \lambda_{2}=\lambda^{2}B_{22}+\lambda^{4}B_{24}\\ \lambda_{3}=\lambda^{3}B_{33}+\lambda^{5}B_{35}\\ \lambda_{4}=\lambda^{4}B_{44}\\ \lambda_{5}=\lambda^{5}B_{55} \end{array}$$ (9)

### Computational domain and boundary conditions

**Fig 5:**
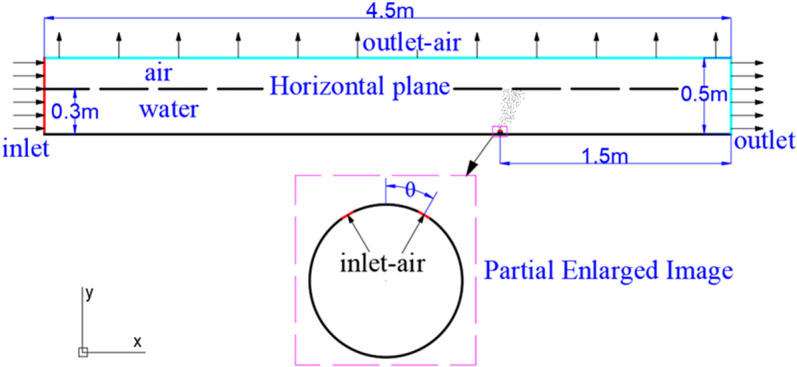
Computational domain modeling diagram.

The computational domain for this study is a rectangular area measuring 4.5 meters in length and 0.5 meters in width. The pipe is positioned in the lower right corner of the domain, 3 meters from the inlet end and 1.5 meters from the outlet end in the *x* direction. The multiphase velocity inlet is set at the far left end of the domain, with the velocity of the second phase set to the water flow speed (*ν*_l_). At the right end of the fluid domain, a diverging outlet boundary is set, and the free surface height and bottom position of the water phase are specified in the open channel. The exhaust pipe is located at the origin, with exhaust holes positioned at the upper left and upper right of the pipe, forming a certain angle and serving as the gas inlet, as shown in Fig 5. Other positions of the exhaust pipe and the bottom of the computational domain are set as wall boundary conditions, while the top of the fluid domain is set as a pressure outlet, with open-air conditions above. The cell registers are used to define the three-phase regions.

### Numerical simulation verification

This study conducted a convergence and independence verification for mesh size and time step in numerical simulations. By comparing the effective oil containment distance from physical experiments and numerical simulations, the most suitable mesh size and time step for numerical simulations were selected.

Mesh independence verification plays a crucial role in numerical simulations. The more refined the mesh, the closer the simulation results are to the real situation. However, a higher number of mesh cells means increased computational workload, longer calculation times, and higher demands on computational resources. Therefore, selecting an appropriate mesh size and time step while maintaining accuracy is essential.

#### Mesh convergence analysis.

**Fig 6:**
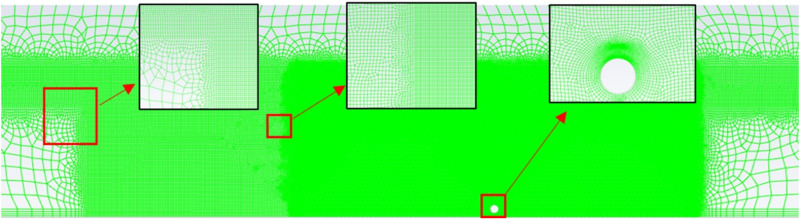
Computational domain mesh division.

The focus of this simulation is on observing the bubble curtain, surface horizontal flow, wave formation, the resulting fluid dynamics phenomena in the domain, and the changes in the oil layer state. Therefore, the mesh is refined at the water surface in the domain. Based on observations from physical experiments, the flow field changes induced by the bubble curtain are mainly distributed upstream of the bubble curtain. Considering that the bubble curtain may shift, the mesh has been locally refined near the exhaust pipe of the pneumatic oil barrier, as shown in Fig 6.

For selecting more appropriate mesh sizes, three different schemes of mesh sizes have been devised, as shown in **Table 3**. The selection of mesh size in the bubble curtain’s active region is crucial for observing the key physical processes of bubbles, including bubble formation, coalescence, and rupture. However, the main goal of this study is to analyze the oil interception performance of the pneumatic oil barrier, particularly how the bubble curtain alters the local water density and affects the horizontal flow to enhance the efficiency of oil spill interception. Therefore, although the shape and volume changes of the bubbles are important factors influencing bubble behavior, this study is more focused on the overall effect of the bubble curtain. According to Arsalan’s findings, [31] the bubble size is related to the nozzle diameter and airflow rate, with larger bubbles forming near the nozzle, often much larger than the nozzle diameter. To reduce the experimental error, a mesh size of 1.5mm and 2mm was chosen for refinement around the bubble curtain in the simulation, based on the nozzle diameter. In order to minimize errors in bubble formation near the nozzle, a local mesh refinement was applied at the nozzle region, as shown in Fig 6.

**Table 3:** Mesh sizes for area refinement.

Sequence	Mesh size in the vicinity of the water surface	Mesh size in the vicinity of the bubble curtain	Total number of meshes
Mesh 1	4mm	1.5mm	207665
Mesh 2	4mm	2mm	134356
Mesh 3	8mm	2mm	110560

**Fig 7:**
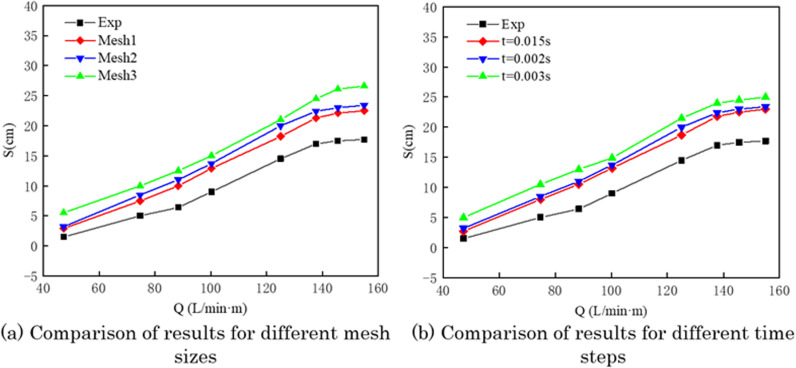
Variation of effective oil-encircling distance under different air supply rates.

To facilitate understanding, the gas flow rate is expressed in terms of the volume of gas released per unit length of the exhaust pipe per minute, denoted as Q (L/min·m). Physical experiments and numerical simulations were conducted for the pneumatic oil barrier with a water flow velocity of 0.07 m/s. The results for the effective oil containment distance from both methods were compared, as shown in Fig 7(a). The results indicate that the effective oil containment distance obtained from the three mesh sizes is close to the experimental measurements and shows similar trends, demonstrating the reliability and accuracy of the numerical simulations. Compared to the experimental results, the velocity values for Mesh 1 and Mesh 2 are closer to the experimental values.

**Fig 8:**
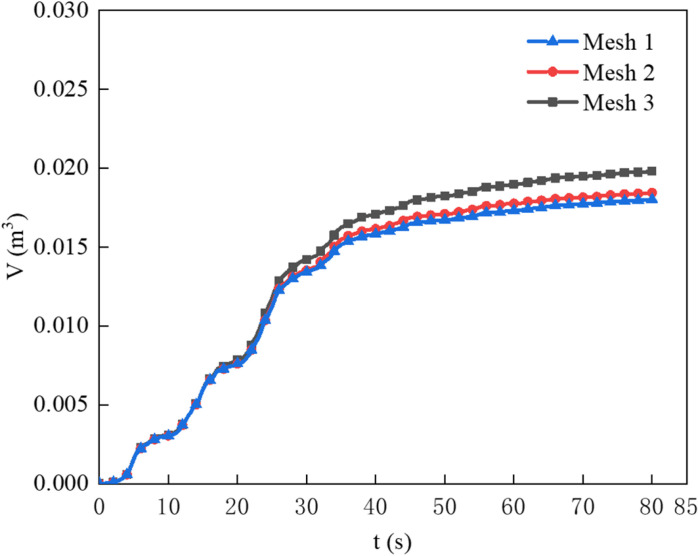
Variation in oil loss across three different mesh sizes.

Fig 8 shows a comparison of oil loss across three mesh sizes under agas flow rate of 74.7 L/min·m. From Fig 8, it can be seen that mesh 1 and mesh 2 are quite similar in terms of oil loss, and the non-dimensional oil volume loss rates for mesh 1, mesh 2, and mesh 3 are calculated as 0.000681s^-1^, 0.000750 s^-1^, and 0.000847 s^-1^, respectively. The numerical differences are almost negligible. However, mesh 2 has a lower computational cost than mesh 1. Given that the results are very similar and mesh 2 offers better computational efficiency, mesh 2 was selected for the numerical simulation calculations. The results from the numerical simulations are slightly larger than the experimental values. This discrepancy may be due to uncertainties in the aeration water sound speed, as reflected in the literature by Jinshu, McClimans, and others, or due to the uneven distribution of bubbles in the longitudinal water domain in the physical experiments, leading to instability in the longitudinal water flow.

#### Convergence analysis of time step size.

In numerical simulations, the choice of time step is also crucial for the accuracy of the results. This study evaluated three different time steps: 0.003 s, 0.002 s, and 0.015 s, while keeping all other numerical settings constant. The results for the effective oil containment distance from these different time steps were compared, as shown in Fig 7(b). Based on the comparison, a time step of 0.002 s was selected for the numerical simulation experiments.

#### Comparison of calculated wave parameters with theoretical values.

**Fig 9:**
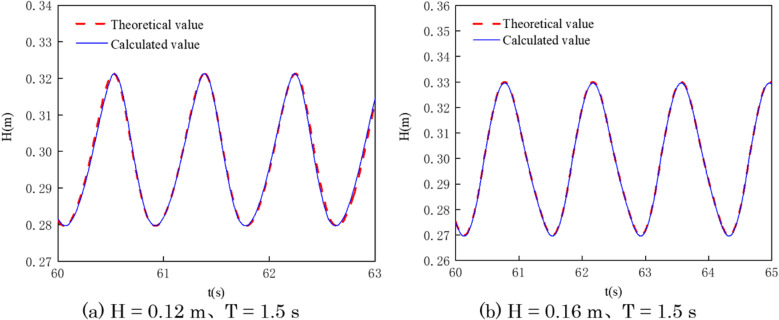
Wave amplitude time series curves.

Based on the convergence analysis of mesh size and time step, a fifth-order Stokes wave model was established under pure wave conditions at the inlet through numerical simulations. Using the known wave height H, wave period T, and water depth d, and combining with the fifth-order Stokes wave theory, the theoretical values of the wave curve were determined. [32] Fig 9 shows the comparison between the theoretical values of wave amplitude time series curves and the waves calculated through numerical simulations for two cases: wave height H=0.12 m with period T=1.5 s, and wave height H=0.16 m with period T=1.5 s. The comparison between calculated and theoretical values indicates a good match, with the maximum error in wave amplitude not exceeding 4%. The wavelength is slightly smaller than the theoretical value, with the single-cycle error not exceeding 0.05 s. The errors in wave amplitude and wavelength are acceptable under the current mesh size conditions. Therefore, it can be verified that the current model is reasonable and suitable for subsequent studies on the oil containment effectiveness of the pneumatic oil barrier.

## Results and discussion

### Experimental and numerical comparison validation

#### Comparison and validation of bubble curtain morphology.

**Fig 10:**
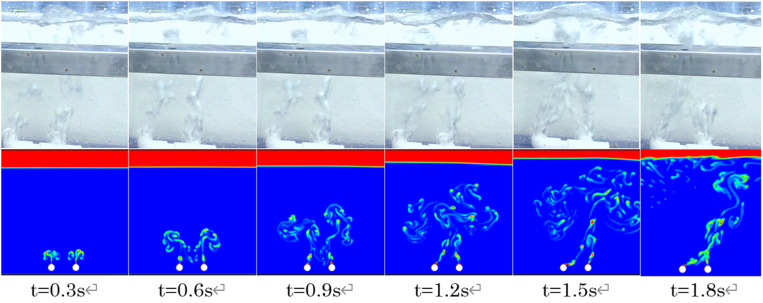
Formation process of the bubble curtain at L = 74mm.

When a pneumatic oil barrier is operating, the shape of the bubble curtain affects the volume of the bubble group in contact with the water. Studies have shown that although the nozzle angles and positions of the gas outlets vary, the bubble curtain changes shape due to the phenomenon of necking. As shown in Fig 10, this study selected a double-pipe pneumatic oil barrier with a maximum distance between pipes L = 74mm. Under a water flow velocity of 0.07 m/s, both physical experiments and numerical simulations were used to compare the shape changes of the bubble curtain at different times. As illustrated, initially, the bubble curtains produced by the two pipes move parallel and upward. At t = 0.9s, the two parallel bubble curtains begin to converge towards the center and undergo necking. At t = 1.2s, the bubble curtains merge into one and move upward. At t = 1.8s, the bubble curtain is displaced due to the water flow. The comparison between numerical simulation results and physical experimental phenomena for the bubble curtain shape at the maximum pipe distance confirms the validity of the numerical simulations.

#### Comparison and validation of waves.

**Fig 11:**
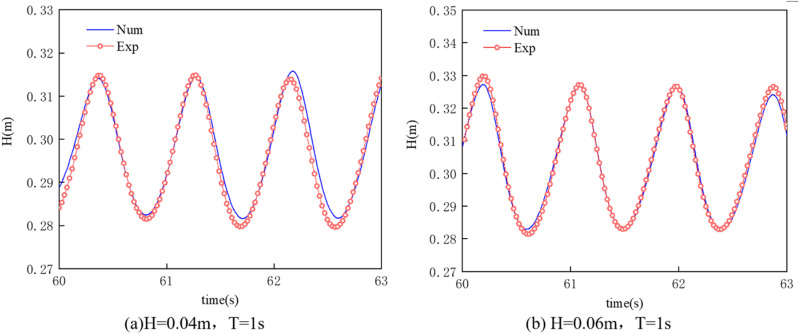
Comparison of amplitude-time curves between physical experiments and numerical simulations.

Using numerical simulations to calculate under the conditions of a gas flow rate of 100.3 L/min·m and a water flow velocity of 0.02 m/s, for waves with H = 0.04 m, T = 1s and H = 0.06 m, T = 1s, the amplitude-time curves of the waves upstream of the pneumatic oil barrier were compared with results measured through physical experiments under the same conditions. The comparison results are shown in Fig 11. The results of the numerical simulation align well with the measurements from the physical experiment, clearly reflecting the consistency in the trends of peak height and period. The amplitude measured in the physical experiment is relatively small, and the experimental wavelength is slightly smaller than the wavelength calculated in the numerical simulation, which may be attributed to three-dimensional effects in the experiment. This validates the correctness of the numerical model.

#### Comparison and validation of oil layer thickness.

**Fig 12:**
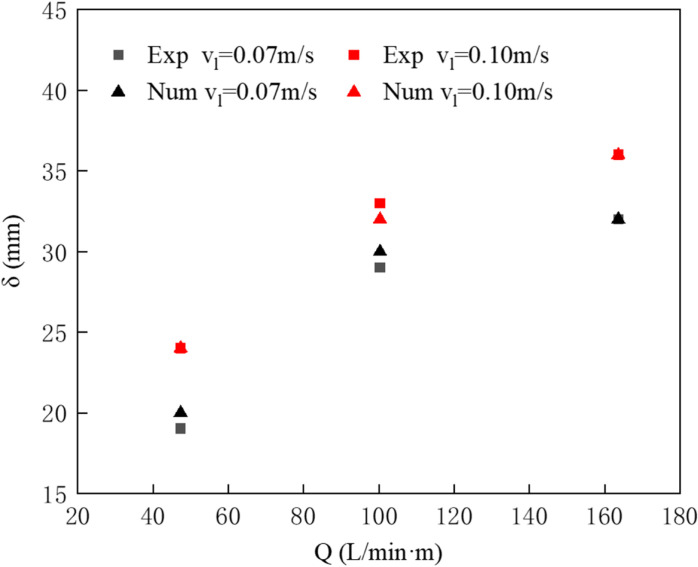
Comparison of oil layer thickness under different flow speed conditions.

Using both physical experiments and numerical simulations, experiments and simulations were conducted on the pneumatic oil barrier with water flow speeds of 0.07 m/s and 0.10 m/s, and an initial oil spill volume of 0.04 m^3^. The oil layer thickness under three different air supply conditions was compared, as shown in Fig 12. The oil layer thickness is denoted as “δ”. Based on observations in both the physical experiment and numerical simulation, the maximum oil layer thickness primarily occurs at the intersection of the water flow and horizontal flow on the water surface, which is also the location where oil droplets often detach. This maximum oil layer thickness is defined as the oil layer thickness in front of the pneumatic oil barrier. As seen in the figure, both the physical experiment and numerical simulation show that as the air supply increases, the oil layer thickness also increases. Moreover, the maximum oil layer thickness in the physical experiment and numerical simulation are quite similar, with the small difference likely attributed to measurement errors caused by the liquid surface shape during the experiment, thereby validating the correctness of the numerical simulation.

#### Oil film morphology under water flow conditions.

**Fig 13:**
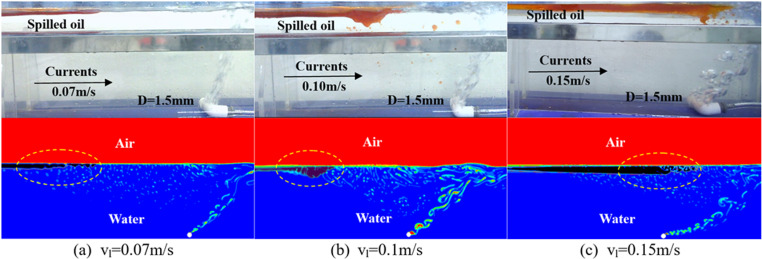
Variation in oil layer morphology under different flow velocities (Top: Physical experiments; Bottom: Numerical simulations).

Through both physical experiments and numerical simulations, the oil interception process and oil layer morphology of the pneumatic oil barrier were examined under three different water flow velocities, and the oil film morphology was compared as shown in Fig 13. At a water flow velocity of 0.07 m/s, the oil layer is longer and thinner with a larger effective oil containment distance, as shown in Fig 13(a). As the water flow velocity increases, the effective oil containment distance decreases, the oil layer length shortens, and local accumulation occurs at the front of the oil layer, increasing its thickness, as shown in Fig 13(b). Further increasing the water flow velocity results in a uniformly thicker oil layer and a shorter effective oil containment distance, as shown in Fig 13(c). While a small amount of oil droplets are produced in physical experiments, they are less noticeable in numerical simulations due to mesh size but can be reflected in the oil interception monitoring curves. The oil layer morphology in numerical simulations closely matches that of physical experiments, validating the effectiveness and accuracy of the numerical simulation results.

### Results analysis

**Fig 14:**
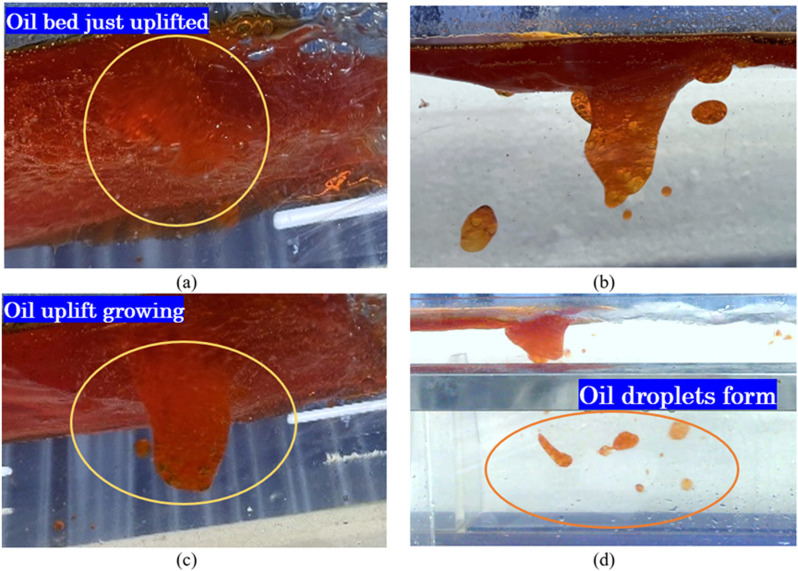
Experimental diagram illustrating the emergence and growth of oil reservoir uplift.

In physical experiments, it was observed that vortices are generated when horizontal flow intersects with water flow. These vortices have a dragging effect on the oil layer, making it prone to forming oil droplets and oil-water mixtures underwater. Fig 14 illustrates the process of oil droplets forming underwater due to the dragging effect of vortices on the oil layer. Fig 14(a), (b) and (c) show thicker oil layer protrusions caused by vortices dragging the oil layer underwater. Fig 14(c) demonstrates that these protrusions are torn and broken, resulting in the formation of oil droplets and oil-water mixture droplets in the water. The primary failure mode of a pneumatic oil barrier during normal operation is entrainment failure, where oil droplets or oil-water mixtures dragged underwater escape the barrier’s interception, resulting in escape through the bubble curtain.

This failure mode is common in the oil interception process of pneumatic oil barriers and increases the probability of entrainment failure. The experiment also revealed that increasing the difference between horizontal flow velocity and water flow velocity increases the likelihood of dragging phenomena. This study analyzes the impact of such occurrences on interception efficiency by examining the quantity of oil intercepted.

**Fig 15:**
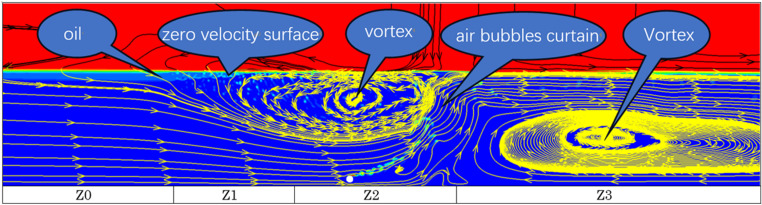
Distribution of streamlines.

In physical experiments, it is challenging to observe the flow field around the pneumatic oil barrier with colorless experimental water, but this can be obtained through numerical simulations. Fig 15 shows the flow streamline distribution of a pneumatic oil barrier with a nozzle diameter of 1.5 mm and a nozzle angle of 30°, under a water flow velocity of 0.07 m/s and a gas flow rate of 125.1 L/min·m. The flow field phenomenon produced by the interaction of water flow can be observed from the streamlines of water molecules. The figure indicates the formation of vortices at both the front and rear ends of the pneumatic oil barrier.

For better analysis, the calculation area is divided into four regions: Z0, Z1, Z2, and Z3. Region Z0 represents the uniform water flow unaffected by the bubble curtain; Z1 is the working area where uniform water flow intersects with the surface horizontal flow, affecting the spread of spilled oil; Z2 is the area at the front of the bubble curtain, where the interaction of uniform water flow and surface horizontal flow generates vortices; Z3 is the area at the rear of the pneumatic oil barrier where vortices are produced. From both physical experiments and numerical simulations, it can be observed that the occurrence of oil droplet detachment, i.e., carryover failure, typically happens at the intersection of the horizontal flow and water flow in the Z1 region, where the oil layer thickness is the greatest. After the two flows converge and move downward, they exert a strong drag on the oil layer. Moreover, as the oil layer thickness increases, the likelihood of oil droplet detachment increases.

When the drag force on the oil layer is large, the combined effect of surface tension and viscosity causes the oil layer to break and form oil droplets. These generated oil droplets follow one of three possible trajectories in the flow field:1. The oil droplet stays at the bottom of the oil layer and eventually merges back with the oil layer after some time. 2. The oil droplet moves with the vortical flow in the Z2 region, follows the flow to the submerged area, and then returns to the oil layer, without passing through or crossing the bubble curtain. 3. The oil droplet moves with the vortical flow in the Z2 region, follows the flow to the submerged area, and then crosses or passes through the bubble curtain, causing an oil retention failure. Due to variations in gas flow rate, water flow velocity, nozzle diameter, and other factors, the speed, thickness, and width of the surface horizontal flow generated by the bubble curtain will vary, causing changes in the size and position of the four regions. The following analysis examines the impact of nozzle diameter, nozzle angle, arrangement, water flow velocity, and waves on the oil interception efficiency of the pneumatic oil barrier.

#### Nozzle parameters ‘effect on oil interception.

This section studies the oil interception effectiveness of the pneumatic oil barrier with nozzle diameters of 1 mm, 1.5 mm, 2 mm, 2.5 mm, and 3 mm, as well as nozzle angles of 0°, 30°, 45°, 60°, and 90°, under conditions of a water flow velocity of 0.07 m/s and a submerged pipe depth of 0.3 m. Additionally, the flow field phenomena around the pneumatic oil barrier are analyzed, with specific operating parameters detailed in **Table 4**.

**Table 4:** Operating parameters table considering the influence of nozzle parameters.

Case	Pipe Diameter	Nozzle Diameter Φ(mm)	Nozzle Spacing d(mm)	Nozzle Angle θ	Pipe Arrangement
A1	DN16	1	15	30°	Single
A2	DN16	1.5	15	30°	Single
A3	DN16	2	15	30°	Single
A4	DN16	2.5	15	30°	Single
A5	DN16	3	15	30°	Single
B1	DN16	1.5	15	0°	Single
B2	DN16	1.5	15	45°	Single
B3	DN16	1.5	15	60°	Single
B4	DN16	1.5	15	90°	Single

First, we analyze the impact of different nozzle diameters on the oil interception effectiveness of the pneumatic oil barrier. According to Bernoulli’s principle, with the same gas supply rate, the gas entrance speed at the nozzle varies with different nozzle diameters; however, when the supply pressure is constant, the gas entrance speed at the nozzle remains the same. To avoid the influence of gas exit speed on the results, this section studies the oil interception effectiveness of pneumatic oil barriers with different nozzle diameters under the same supply pressure. The supply pressure is represented using a pressure multiplier (the depth of the gas supply pipe), denoted as P₀.

**Fig 16:**
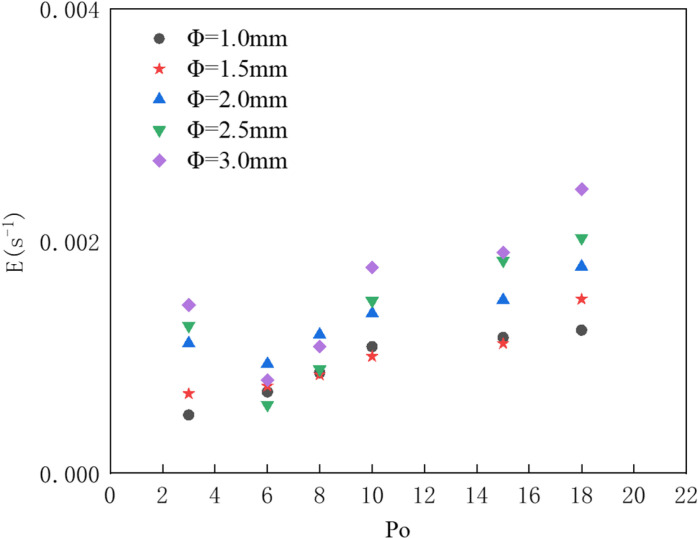
Dimensionless oil volume loss rate for different nozzle diameters.

Fig 16 illustrates the calculation of oil interception loss through numerical simulation. The product of the actual spill volume (m^3^) and the dimensionless oil volume loss rate under the same environmental conditions can be used to predict the actual oil volume passing through. As the supply pressure increases, the dimensionless oil spill volume loss rate increases. Under constant supply pressure, increasing the nozzle diameter of the pneumatic oil barrier also results in an increased dimensionless oil volume loss rate, with larger supply pressures causing a more significant increase in the loss rate. The figure indicates that pneumatic oil barriers with nozzle diameters of 1 mm and 1.5 mm exhibit similar and favorable oil interception effectiveness.

**Fig 17:**
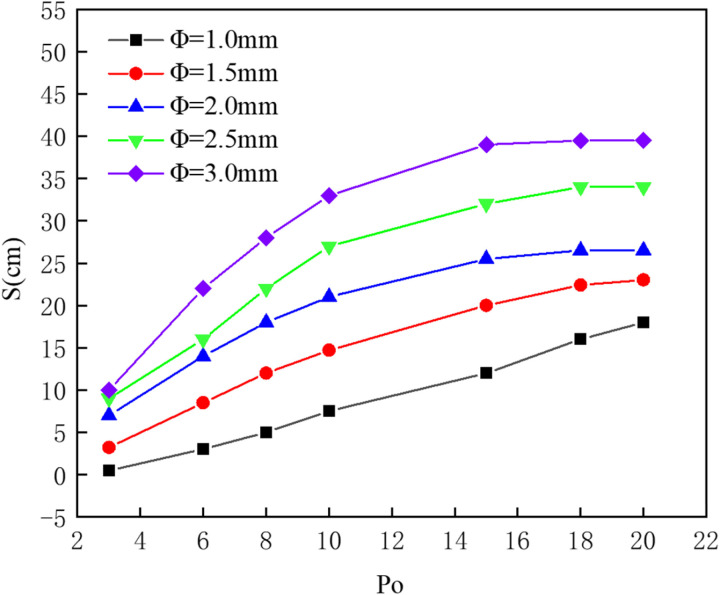
Comparison of effective containment distance for different nozzle diameters.

Fig 17 presents a comparison of effective containment distances for five different nozzle diameters. The figure shows that under the same pressure conditions, an increase in nozzle diameter leads to a longer effective containment distance. As the supply pressure increases, larger nozzle diameters exhibit a greater rate of increase in effective containment distance, with barriers featuring larger nozzles more easily reaching saturation (beyond which increases in pressure do not result in further increases in effective containment distance). The data indicate that the pneumatic oil barrier with a 1.5 mm nozzle diameter has a greater effective containment distance than that with a 1 mm nozzle diameter. Both physical experiments and numerical simulations have observed that as the nozzle diameter increases, oil droplets are more easily dragged underwater at the leading edge of the oil layer. To explain this phenomenon, numerical simulations were conducted to calculate the flow trajectory distribution for the five different nozzle diameters, allowing observation of the flow field phenomena around the pneumatic oil barrier.

**Fig 18:**
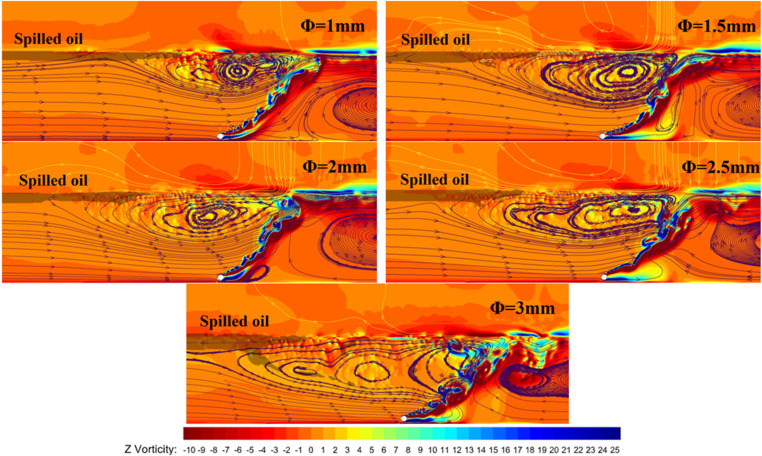
Flow trajectory distribution and vorticity map for different nozzle diameters.

This comparison focuses only on the flow field under a pressure multiplier of 10. Fig 18 illustrates the flow trajectory distribution and vorticity map of the water surrounding the pneumatic oil barrier, calculated through numerical simulations at a supply pressure of ten times the pressure at the water depth, with a water flow velocity of 0.07 m/s. From the distribution of the flow trajectories, it is evident that when the bubble curtain carries surrounding water to the surface, it generates a laterally spreading surface horizontal flow. The upstream horizontal flow moves in the opposite direction to the water flow; upon intersecting with it, the combined forces cause downward movement, creating a vortex at the front of the bubble curtain. The trajectories close to the water surface indicate the presence of this surface horizontal flow.

As the nozzle diameter increases, the width of the vortex also increases, and the length of the flow trajectories near the water surface becomes longer, suggesting that larger nozzle diameters can generate greater upward kinetic energy, thereby increasing the width of the surface horizontal flow. This explains the increase in effective containment distance. However, as the effective containment distance increases, the length of the oil layer continues to decrease while its thickness increases. When the nozzle diameter reaches 2.5 mm, a small number of oil droplets are observed being dragged to the surface; some follow the flow field back upstream, while others penetrate the bubble curtain, leading to containment failure. Moreover, as the nozzle diameter increases, the flow trajectories near the water surface transition from straight lines to wavy patterns, indicating that larger nozzle diameters exacerbate the disturbances caused by the bubble curtain on the horizontal flow. These disturbances can also be observed in physical experiments.

In the figure, the colors represent the magnitude of the vorticity. Orange indicates zero vorticity, while the vorticity increases with the redness of the color in the clockwise direction, and decreases in the counterclockwise direction with the vorticity transitioning from yellow to blue. The greater the difference in color from red, the higher the vorticity. Regions with higher vorticity generally indicate stronger rotational motion of the fluid. From the figure, it can be observed that at the junction of the two flows, as the nozzle diameter increases, the difference between the red and yellow colors becomes more pronounced, and generally, yellow and red appear together, with yellow on the left and red on the right. For example, with nozzle diameters of 2.5mm and 3mm, the bubble curtain at the junction of the surface horizontal flow and water flow shows a significant difference in vorticity color, indicating potential disturbances. The fluid motion in this area exhibits clear rotation and shear forces, making it prone to tearing the oil layer and forming oil droplets. At the junction of the two flows, due to the high flow velocity of the horizontal flow and the resulting disturbances in the water flow, the larger vorticity at the front of the oil layer leads to instability and the generation of oil droplets. Therefore, while increasing the effective oil retention distance, reducing disturbances and ensuring a more uniform distribution of vorticity at the junction point are essential for efficient oil spill control.

Combining the analysis of effective containment distance and oil interception volume for the pneumatic oil barriers, it is evident that while both the 1 mm and 1.5 mm nozzle diameters exhibit good oil interception effectiveness, the barrier with a 1.5 mm nozzle diameter has a greater effective containment distance. Therefore, it can be concluded that the pneumatic oil barrier with a 1.5 mm nozzle diameter offers the best oil interception performance. When the nozzle diameter is larger, the larger bubbles tend to burst as they rise to the water surface, intensifying surface disturbances. If the effective containment distance is too small, these disturbances can adversely affect the stability of the oil layer, resulting in an increase in the volume of spilled oil.

**Fig 19:**
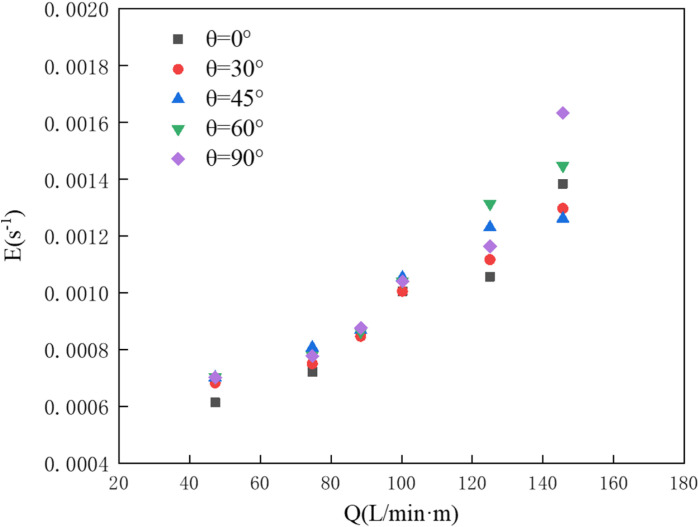
Dimensionless oil volume loss rate for different nozzle angles.

Next, under a water depth of 0.3 m and a flow velocity of 0.07 m/s, we examine the impact of different nozzle angles on the oil interception effectiveness of the pneumatic oil barrier. Fig 19 displays the dimensionless oil volume loss rates calculated through numerical simulations for pneumatic oil barriers with various nozzle angles. As the gas supply increases, the dimensionless oil volume loss rate also rises. The similar loss rates across different nozzle angles indicate comparable oil interception effectiveness.

**Fig 20:**
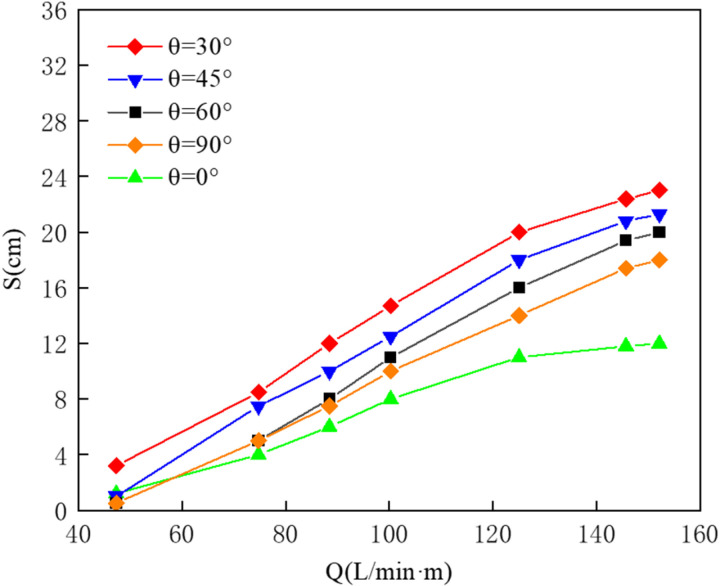
Analysis of effective containment distance for different nozzle angles.

Fig 20 presents a comparison of effective containment distances for different nozzle angles. Under the same gas supply conditions, the pneumatic oil barrier with a 0° nozzle angle exhibits a smaller effective containment distance compared to others, while the barrier with a 30° nozzle angle shows a larger effective containment distance. As the gas supply increases, the effective containment distances for all five nozzle angles also rise. Upon reaching saturation, the barrier with a 30° nozzle angle achieves a greater effective containment distance than the others.

In the physical experiments, it was found that although the gas entrance velocities differ in direction for different nozzle angles, the bubble curtain generated by the pneumatic oil barrier rapidly converges into a coherent bubble curtain due to the phenomenon of necking after the gas is expelled, making it difficult to observe distinct differences in the shapes of the bubble curtains. Furthermore, no significant differences in the oil layer states of the pneumatic oil barriers with different nozzle angles could be observed under the same gas supply conditions. To explain the variations in effective containment distance, a flow field analysis will be conducted using numerical simulation.

**Fig 21:**
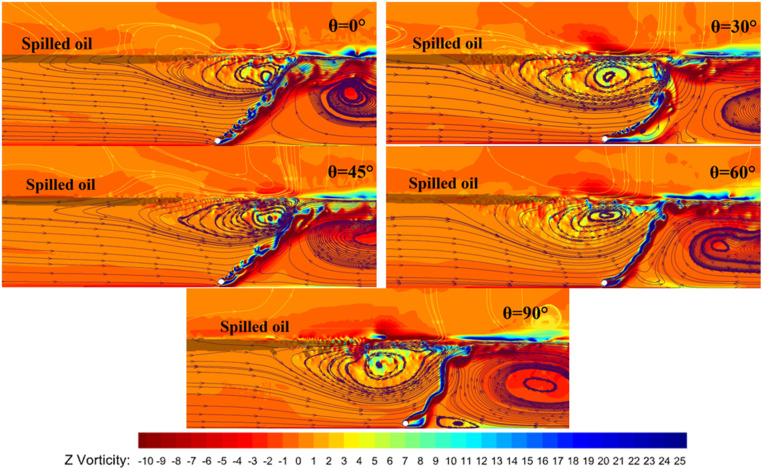
Flow trajectory distribution and vorticity map for different nozzle angles.

Fig 21 presents the flow trajectory distribution and vorticity maps calculated for pneumatic oil barriers with five different nozzle angles under a water flow velocity of 0.07 m/s and a gas supply rate of 125.1 L/min·m. This analysis focuses solely on the flow field conditions at this specific gas supply rate. As illustrated, as the nozzle angle increases from 30° to 90°, the flow trajectories near the water surface shorten, resulting in a reduced effective containment distance, with the flow trajectory at a 0° angle being the shortest. From the figure, it is evident that the vortex around the pneumatic oil barrier with a 30° nozzle angle is elongated, and the flow trajectories near the water surface remain close to the surface, indicating that the surface horizontal flow is broad, flat, and stable, which contributes to a larger effective containment distance. As the nozzle angle increases, the flat horizontal flow shortens, leading to a decreased effective containment distance.

From the figure, it can be seen that the red and yellow regions representing vorticity appear in pairs. As the nozzle diameter increases, the color difference in the high vorticity regions at the horizontal flow location becomes more pronounced, and the high vorticity area at the front of the oil layer extends to deeper water depths. Compared to other nozzle angles, in the vorticity map for the nozzle angle of 90°, the color difference in the vorticity at the front of the oil layer is the largest, and it affects deeper water regions. The excessive vorticity in this area generates shear forces, causing protrusions in the oil layer to tear and form small oil droplets. Although the leakage is not obvious, this indicates that the flow field motion causes the small oil droplets to circulate in front of the bubble curtain, without resulting in the failure of the oil retention mechanism. Although the leakage for the pneumatic oil barrier at a 90° angle is minimal, the oil layer becomes unstable and is easily disturbed, making it susceptible to leakage.

Therefore, combining the above analysis, while the oil interception effectiveness of the barriers with different nozzle angles is similar, the barrier with a 30° nozzle angle exhibits a greater effective containment distance. Thus, we conclude that among the five nozzle angles tested, the pneumatic oil barrier with a 30° nozzle angle demonstrates the best oil interception performance. In comparison with actual applications, considering the above research results, the oil retention effect of the bubble curtain is optimal when the nozzle diameter is 4.5 mm and the nozzle angle is 30°. Under this configuration, the bubble curtain generates a relatively mild horizontal flow, and the disturbances on the water surface have a minimal impact on the surface oil layer, leading to the best oil retention effect. Of course, the choice of nozzle diameter is not limited to 4.5 mm; the larger the nozzle diameter, the greater the effective oil retention distance and the stronger the anti-interference ability to respond to emergency situations. A larger diameter can be selected based on the actual environment to improve the oil retention effect of the bubble curtain.

#### Effect of arrangement on oil interception.

The pneumatic oil barriers are arranged in a parallel configuration with two exhaust pipes, forming a dual-pipe layout. The dual-pipe arrangement can impact a larger water area during gas release. Therefore, this section will investigate the effects of single-pipe and dual-pipe arrangements on oil interception effectiveness, with specific operating conditions outlined in **Table 5**. The distance between the two exhaust pipes in the dual-pipe arrangement is represented as L (mm). Since the number of nozzles in the dual-pipe arrangement is twice that of the single-pipe arrangement, this section will analyze the impact of the arrangement style on oil interception effectiveness under the same pressure multiplier conditions, as detailed below:

**Table 5:** Parameters for exhaust pipe arrangement methods.

Case	Pipe Diameter	Nozzle Diameter Φ(mm)	Nozzle Spacing d(mm)	Nozzle Angle(θ)	Arrangement Method
G1	DN16	1.5	15	30°	Single
G2	DN16	1.5	15	30°	Dual L=28mm
G3	DN16	1.5	15	30°	Dual L=44mm
G4	DN16	1.5	15	30°	Dual L=63mm
G5	DN16	1.5	15	30°	Dual L=74mm

**Fig 22:**
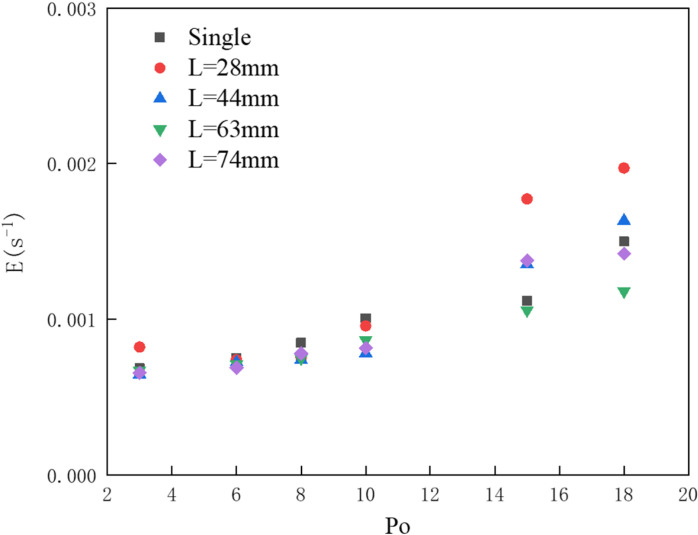
Dimensionless oil volume loss rate.

As shown in Fig 22, the dimensionless oil volume loss rates for pneumatic oil barriers with different arrangements indicate that as the gas supply pressure increases, the dimensionless oil passage rate also increases, albeit at varying rates. Compared to the single-pipe deployed barriers, those with a pipe distance of 28 mm show a greater increase, while those with a 63 mm distance exhibit a smaller increase. Even at a pressure multiplier of 18, they maintain good oil interception effectiveness.

**Fig 23:**
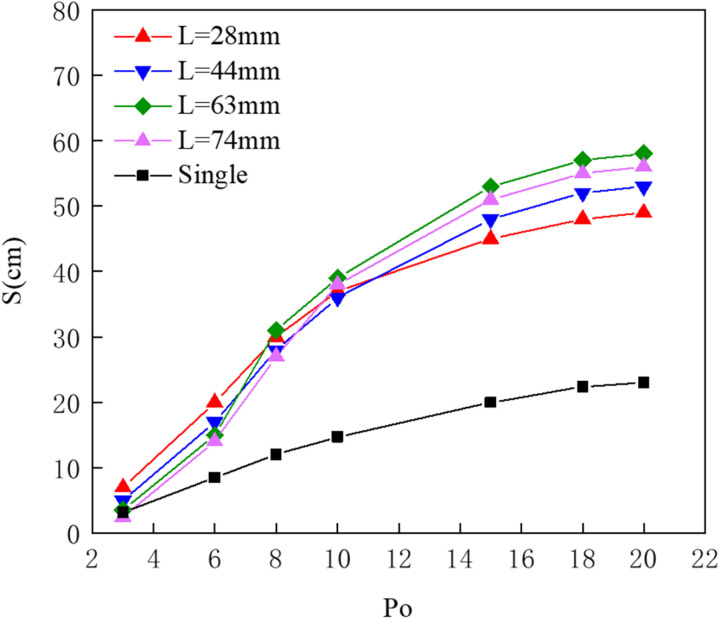
Comparison of effective containment distance for single-pipe and dual-pipe arrangements.

Fig 23 presents a comparison of effective containment distances under the same pressure multiplier conditions. With consistent gas supply rates for both arrangements at the same pressure, it is clear that the dual-pipe configuration provides a greater effective containment distance than the single-pipe arrangement. As the pressure multiplier increases from 3 to 15, the effective containment distance for the dual-pipe arrangement grows more rapidly. Upon reaching saturation, the effective containment distance for the dual-pipe arrangement far exceeds that of the single-pipe setup, likely because the dual-pipe configuration generates a broader and thicker horizontal flow by impacting more water.

**Fig 24:**
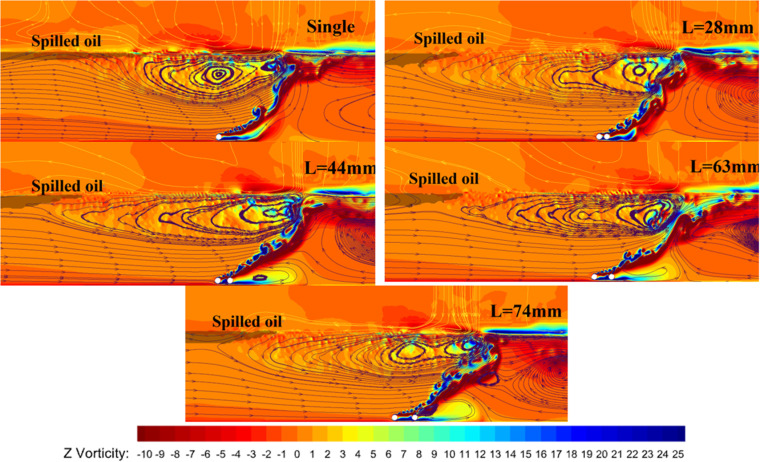
Flow trajectory distribution and vorticity map for single and double pipe arrangements.

To analyze the reasons behind the differing effective containment distances under the same pressure conditions, numerical simulations of the flow fields were conducted. Fig 24 illustrates the flow trajectory distribution and vorticity maps for both single-pipe and dual-pipe arrangements under a water flow velocity of 0.07 m/s and a pressure multiplier of 15. The longer and straighter flow trajectories near the water surface indicate that the dual-pipe arrangement influences more water, resulting in a thicker and wider horizontal flow, thus leading to a greater effective containment distance. From the colors representing the vorticity in the figure, it can be observed that the bubble curtain with a double-pipe arrangement has a smaller red-yellow color difference at the intersection of the two flows compared to the single-pipe arrangement. The color difference is smaller and more evenly distributed. This indicates that the vorticity in this region is smaller, and the shear force generated is also lower, which helps maintain better stability of the oil layer. It suggests that although the double-pipe arrangement of the bubble curtain has a larger effective oil retention distance than the single-pipe arrangement, the smaller disturbances and lower vorticity in the small vortex flow generated by the double-pipe arrangement result in a better oil retention effect.

Overall, the analysis confirms that the dual-pipe arrangement of pneumatic oil barriers offers superior oil interception performance, particularly when the distance between pipes is 63 mm. In environments prone to oil layer instability, utilizing dual-pipe arrangements is advisable. In practical applications, the double-pipe arrangement of the bubble curtain has stronger anti-interference capability compared to the single-pipe arrangement, making it more suitable for environments prone to unpredictable disturbances. When selecting the double-pipe arrangement for the bubble curtain, a pipe spacing of about 189mm provides a good oil retention effect. In this configuration, the horizontal flow generated by the bubble curtain is insufficient to cause the oil layer to form droplets, thus reducing the likelihood of entrainment failure. However, the use of the double-pipe arrangement requires a larger air supply, which places higher demands on the air compressor’s storage capacity and compression speed. Depending on the unpredictability of the environment, it may also be necessary to choose a higher air supply pressure.

#### Water flow velocity affects oil containment effectiveness.

**Fig 25:**
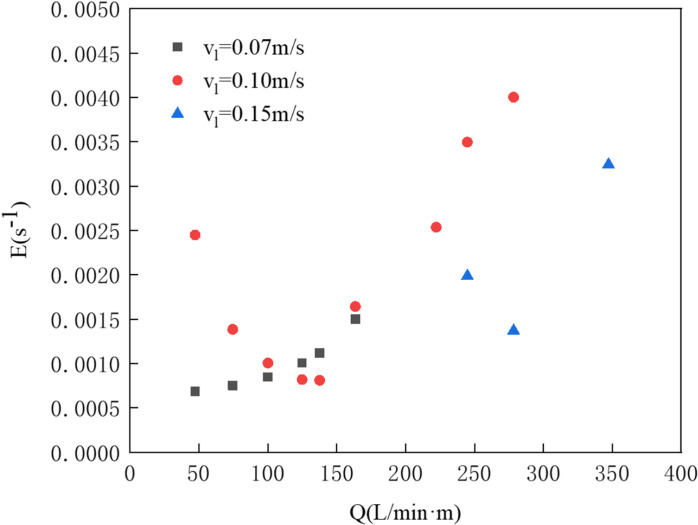
Dimensionless oil spill volume loss rate.

The physical experiments and numerical simulations were conducted for pneumatic oil barriers under water flow velocities of 0.07 m/s, 0.10 m/s, and 0.15 m/s. Using numerical simulations, the dimensionless oil volume loss rates were calculated for an initial oil volume of 0.04 m^3^ under these three flow conditions, as shown in Fig 25. The results indicate that with increased gas supply, the oil volume passage rate at 0.07 m/s increases, with a saturation point reached at a dimensionless oil volume loss rate of 0.00153. At 0.10 m/s, the loss rate initially decreases before rising again, reaching a minimum of 0.00079 at a gas supply of 137.8 L/min·m, which is still higher than the rate at 0.07 m/s with 473 L/min·m. When the flow speed increases to 0.15 m/s, the barrier can intercept oil but experiences a higher volume passage rate.

**Fig 26:**
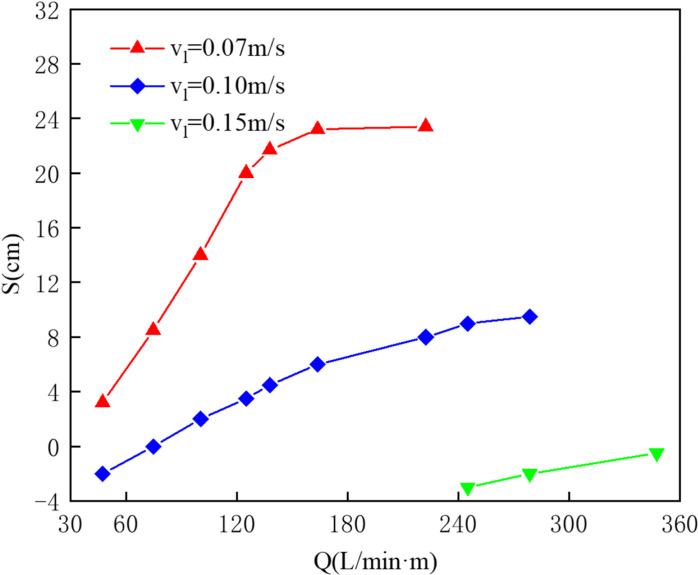
Comparison of effective oil-encircling distance results at different water flow velocities.

Fig 26 compares the effective containment distances at different flow speeds. With the same gas supply, increasing water flow velocity leads to a reduction in effective containment distance; for instance, at a supply of 125.1 L/min·m, the effective containment distance decreases from 20 cm at 0.07 m/s to 2 cm at 0.10 m/s. Increasing gas supply improves the interception effectiveness across all flow speeds. Notably, at a flow speed of 0.15 m/s, when the gas supply is below 245 L/min·m or above 347.4 L/min·m, the pneumatic oil barrier fails to form a gas bubble curtain to effectively intercept oil, which is why effective containment distances are not displayed in Fig 26. Between these two supply values, effective containment distances become negative. As gas supply increases, the oil layer moves downstream, increasing both effective containment distance and stability. The study reveals that as water flow speed rises, the gas bubble curtain produced by the pneumatic oil barrier shifts further downstream, causing the oil layer to compress and thicken.

**Fig 27:**
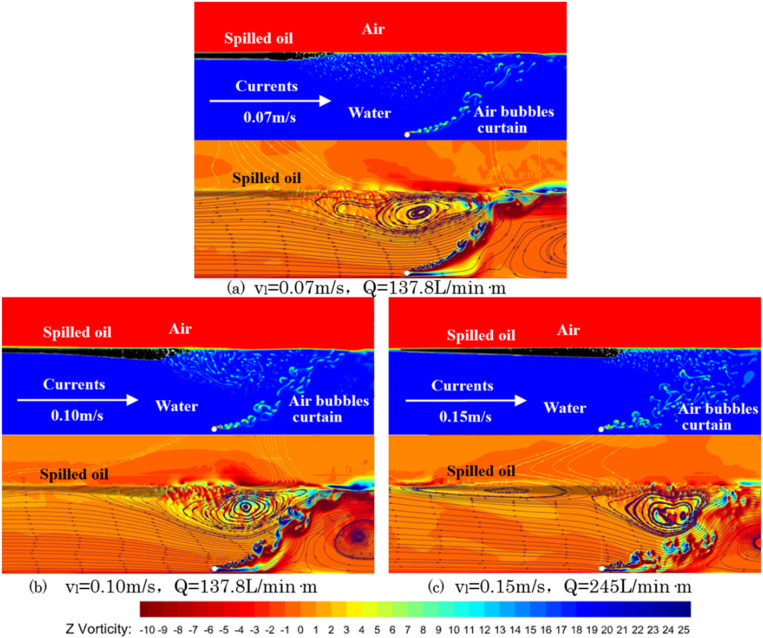
Density distribution and flow trajectory distribution with vorticity map.

As shown in Fig 27, the upper section illustrates the simulation results under three different flow speeds, while the lower section presents the streamlines and vorticity distribution. The simulation indicates that as the water flow speed increases, the gas bubble curtain shifts further downstream and adopts an umbrella-like distribution, resulting in a reduced oil layer length and an increased thickness. From the flow trajectory distribution and vorticity map shown below, it can be seen that the colors represent the vorticity magnitude of the bubble curtain under different flow speeds. The greater the color difference between red and yellow, the larger the shear force; the darker the color, the greater the vorticity. The chart indicates that as water speed increases, the color in the horizontal flow region becomes deeper, and the color difference within this region becomes larger. This suggests that the increase in water speed causes both the vorticity and the vorticity gradient to grow. The high vorticity regions produce strong shear forces that can impact the stability of the oil layer, thereby increasing the likelihood of entrainment failure.

In summary, the pneumatic oil barrier can effectively intercept oil spills in a uniform water flow environment at 0.15 m/s when supplied with an appropriate gas quantity. As the flow speed increases, the effective containment distance also increases, along with an increase in oil layer thickness. Therefore, increasing the gas supply appropriately as water flow speed rises can help reduce the volume of oil lost. In practical applications, the bubble curtain oil containment system can effectively intercept oil spills in water flow environments with flow speeds less than 0.26 m/s. However, it is important to control factors such as exhaust volume and lower wind speeds to ensure the effectiveness of the system in intercepting the oil spill. Therefore, in actual applications, when the water flow speed is equal to or greater than 0.26 m/s, the use of a bubble curtain oil containment system for oil spill interception is not recommended. Conversely, when the water flow speed is less than 0.26 m/s, the bubble curtain oil containment system can provide better interception performance.

#### Impact of waves on oil containment effectiveness.

This section investigates the oil interception performance of the pneumatic oil barrier under coupled wave and flow conditions, with a water flow speed of 0.02 m/s and wave parameters as outlined in **Table 6**. Due to the turbulence at the water surface, the effective containment distance of the pneumatic oil barrier varies. Therefore, this section does not provide specific values for the effective containment distance but focuses on studying the interception effectiveness in terms of the dimensionless oil volume loss rate and the effects of waves and flow field velocity distribution.

**Table 6:** Wave environment parameter conditions.

Case	Water Flow Velocity v_1_(m/s)	Wave Height H(m)	Wave Period T(s)
W1	0.02	0.04	0.75
W2	0.02	0.04	1
W3	0.02	0.04	1.5
W4	0.02	0.06	0.75
W5	0.02	0.06	1
W6	0.02	0.06	1.5

**Fig 28:**
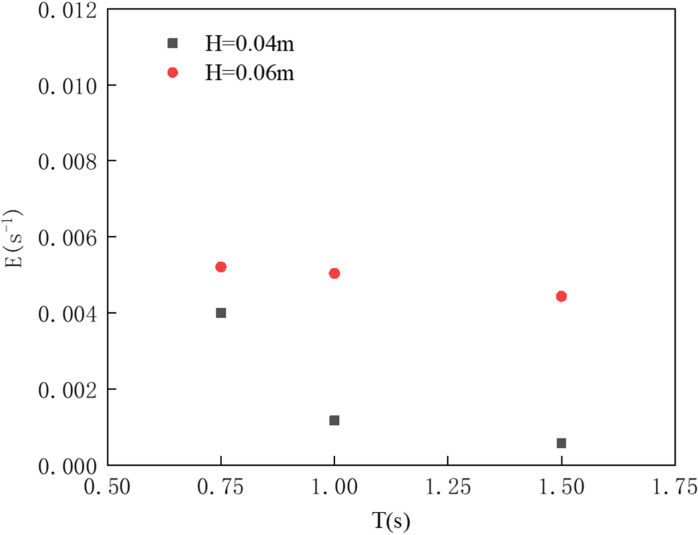
Dimensionless oil volume loss rate.

As shown in Fig 28, the comparison of the dimensionless oil volume loss rate results for the oil boom under different wave parameters is presented. The figure indicates that, with a constant wave period, an increase in wave height leads to a higher dimensionless oil volume loss rate. Conversely, with a constant wave height, increasing the wave period results in a decrease in the dimensionless oil volume loss rate. When the wave period exceeds 1 second, the rate of decrease in the dimensionless oil volume loss rate slows down.

**Fig 29:**
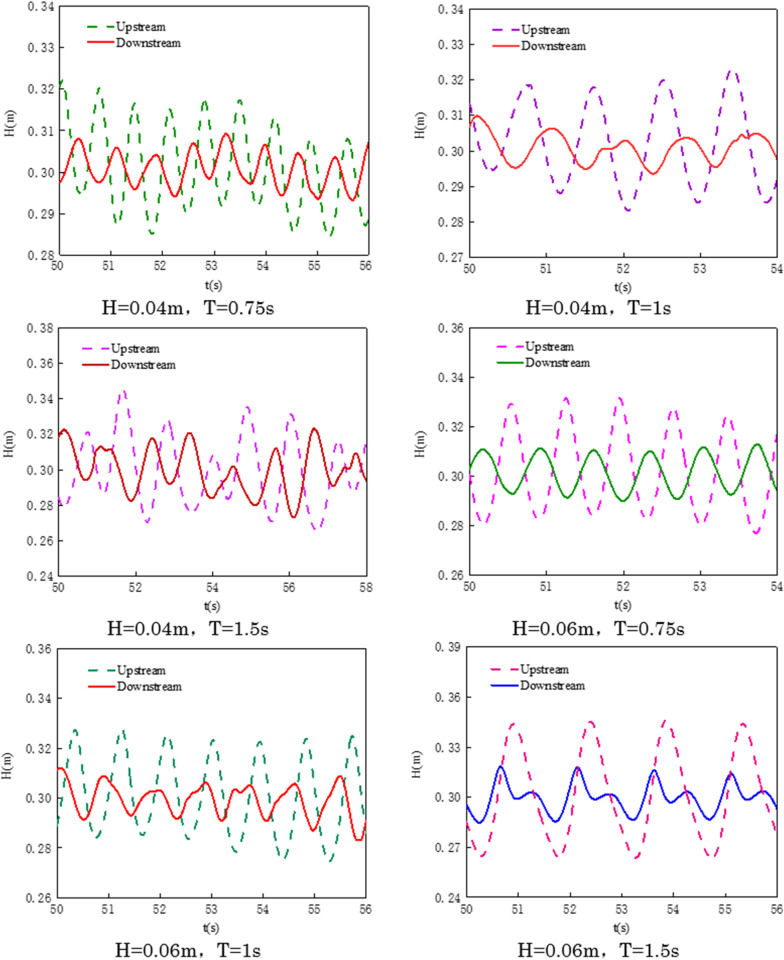
Time series curves of wave amplitude upstream and downstream of the air curtain oil containment boom.

As shown in Fig 29, the wave time history curves are obtained through numerical simulation to monitor the wave variations upstream and downstream of the air curtain oil containment boom. The figure shows that the air curtain oil barrier not only intercepts oil spills but also reduces wave amplitude. When using two barriers at a certain distance, it can help with oil containment. When the wave period is less than 1 second, the waves can cause oil droplets to form, increasing the likelihood of entrainment failure.

**Fig 30:**
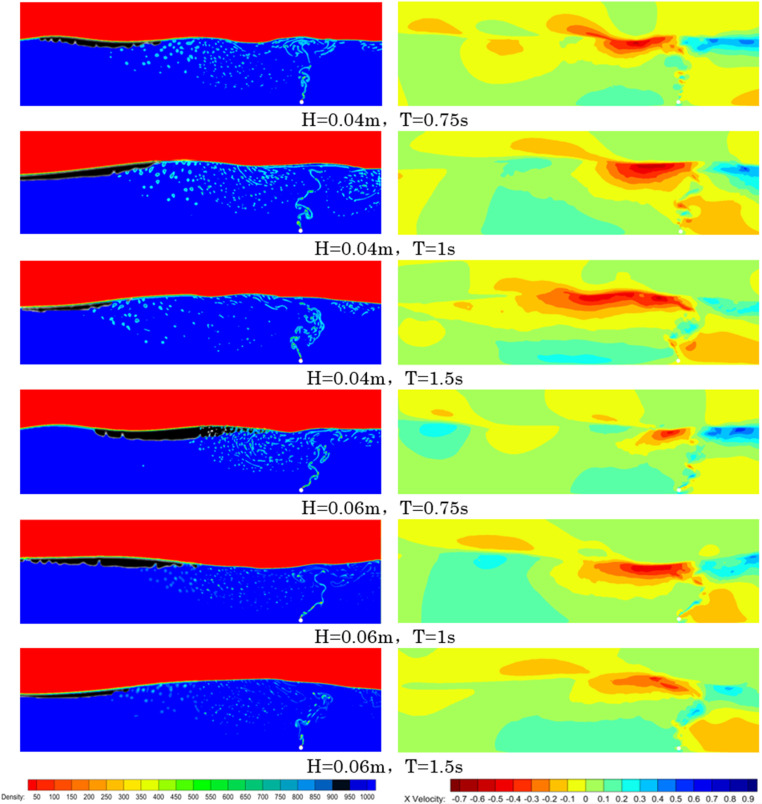
Oil containment simulation and velocity cloud maps.

Although waves can alter the effective oil containment distance, the study found that a shorter wave period leads to an increased effective oil containment distance. As shown in Fig 30, which displays simulation results and velocity cloud maps under six different wave conditions, it can be observed that with smaller wave periods, the oil layer is shorter and thicker, resulting in a reduced effective oil containment distance. From the velocity cloud map on the right, it is evident that for smaller wave periods, the distribution of horizontal flow is shorter. Under the same wave period conditions, increasing the wave height reduces the effective oil containment distance, narrows the width of the horizontal flow, and increases the amount of oil loss. In practical applications, it is not recommended to use the bubble curtain oil containment system alone for oil spill interception in wave environments. It can, however, be combined with a boom-type oil containment system for more effective oil spill interception. The bubble curtain not only has oil retention capabilities, but its bubble curtain also plays a role in reducing wave amplitudes and can be deployed upstream of the boom-type oil containment system. In wave conditions, the bubble curtain becomes unstable, and the interaction between the generated horizontal flow and the waves intensifies surface disturbances, reducing the stability of the oil layer. While it can still intercept the oil spill, the likelihood of entrainment failure increases.

## Conclusion

This paper adopts a combined approach of physical experiments and numerical simulations to analyze the oil interception effect of pneumatic oil barriers under uniform flow and wave action, considering both oil loss situations and effective oil containment distances. Factors such as nozzle diameter, nozzle angle, single pipe and double pipe arrangements, water flow velocity, and wave effects on the performance of pneumatic oil barriers were studied, and the differences in effective oil containment distance were analyzed using flow field distributions.

The following conclusions were drawn:

(1). The main failure mode of pneumatic oil barriers during normal operation is entrainment failure; maintaining the stability of the oil layer is essential for improving the oil interception success rate of pneumatic oil barriers.
(2). Although increasing the nozzle diameter of the pneumatic oil barrier can enhance the effective oil containment distance, it can also compromise the stability of the oil layer, increasing the likelihood of floating oil escape. Choosing an appropriate nozzle diameter is crucial for enhancing the oil control capability of pneumatic oil barriers. A pneumatic oil barrier model with a nozzle diameter of 1.5 mm deployed in water with a depth of 0.3 m can meet oil containment needs while maintaining good oil layer stability, providing a reference for the design of pneumatic oil barriers.
(3). The variation in effective oil containment distance indicates that the occurrence of the contraction phenomenon minimizes the impact of nozzle angle on the oil containment characteristics of pneumatic oil barriers.
(4). Under the condition of maintaining the same gas pressure in the gas delivery pipe, the double pipe arrangement of pneumatic oil barriers exhibits better oil interception performance. This study provides a reference for the more economical use of pneumatic oil barriers in practical environments. Choosing an appropriate distance between pipes in the double pipe arrangement can achieve better oil interception effects.
(5). In uniform flow, pneumatic oil barriers perform well in low flow velocities (below 0.15 m/s); selecting an appropriate gas supply amount is essential for intercepting oil in a water flow velocity of 0.15 m/s.
(6). Ensuring a uniform distribution of vorticity in the water area at the front of the oil layer is a prerequisite for maintaining the stability of the oil layer.
(7). Waves can affect the horizontal flow of pneumatic oil barriers; increasing wave height reduces the width of horizontal flow and the effective oil containment distance; decreasing wave period also reduces the width of horizontal flow and the effective oil containment distance. Waves can impact the stability of the oil layer; increased wave height and decreased wave period can compromise oil layer stability and increase the likelihood of entrainment failure.

## The mechanisms of failure modes and future research recommendations

**Fig 31:**
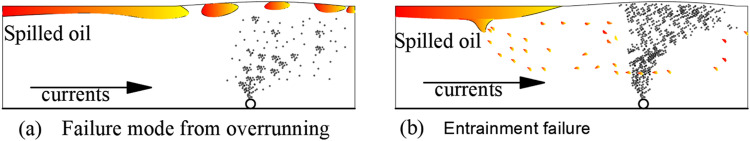
Schematic diagram of oil retention failure modes.

Based on the above analysis, when the air curtain oil boom device is functioning properly, there are primarily two types of failure modes: overtopping failure (as shown in Fig 31(a)) and entrainment failure (as shown in Fig 31(b)). Overtopping failure typically occurs when the air bubble curtain is unstable, the gas supply is insufficient, or the water flow speed is too high. The main cause is that the generated horizontal flow is insufficient to prevent the spread of the spilled oil. To mitigate this failure, strategies such as increasing the gas supply, enlarging the nozzle aperture, choosing an appropriate nozzle angle, or reducing the water flow speed can be employed. The most common failure mode is entrainment failure, which often occurs when there is significant surface disturbance, a large difference between the water flow speed and the horizontal flow speed, or when the oil layer is thick. The main cause is that the buoyancy of the bubble curtain is too high, causing large disturbances on the water surface or generating horizontal flow with excessive speed. In a wave environment, this failure mode occurs because, on one hand, larger wave heights or shorter wave periods increase the interaction between the oil layer and water, thereby increasing the likelihood of oil layer detachment; on the other hand, the horizontal flow generated is opposite to the direction of wave propagation, and the interaction between the waves and horizontal flow further amplifies disturbances on the water surface and oil layer. To mitigate this failure, measures such as reducing the gas supply, narrowing the nozzle aperture, or increasing the volume of water influenced by the bubble curtain (e.g., by increasing the number of exhaust pipes) can be taken.

However, based on the above research on the oil retention effectiveness of the air curtain boom and the analysis of failure mechanisms, I believe there is still a significant amount of research to be done in the future. This study is limited by the experimental environment, utilizing scaled physical experiments and numerical simulations. Future research can further refine, validate, and expand this study through experiments at a larger scale or field trials, combined with numerical simulations. In real sea conditions, waves are affected by tidal forces, wind speed, wind direction, ocean depth, and other factors, making the wave environment highly variable and complex. This study only addressed the effect of oil retention under regular wave conditions, without investigating the performance of the air curtain boom under irregular waves. Future research can expand to study oil retention in irregular waves, co-directional or counter-directional wave currents, and other complex environments. Factors such as wind speed, wind direction, temperature, and humidity also have a significant impact on the spread of spilled oil, but there is currently a lack of systematic analysis on these factors. Moreover, during an oil spill, to increase the oil retention rate of various booms, coagulating agents are often sprayed on the floating oil to increase its viscosity and suppress its spread. This method may reduce or prevent entrainment failure in the air curtain boom, so research on oil spill interception at different viscosities could also become a key focus for future studies. Additionally, the nozzle aperture geometry, spacing, and the practical design of nozzle devices influence the oil retention effectiveness and fluid dynamics of the air curtain boom. Research in this area, though relatively limited, could provide valuable insights for future improvements and design recommendations for air curtain booms.
